# Pathogenic and Transmission Potential of Wildtype and Chicken Embryo Origin (CEO) Vaccine Revertant Infectious Laryngotracheitis Virus

**DOI:** 10.3390/v13040541

**Published:** 2021-03-24

**Authors:** Ana Perez-Contreras, Catalina Barboza-Solis, Shahnas M. Najimudeen, Sylvia Checkley, Frank van der Meer, Tomy Joseph, Robin King, Madhu Ravi, Delores Peters, Kevin Fonseca, Carl A. Gagnon, Davor Ojkic, Mohamed Faizal Abdul-Careem

**Affiliations:** 1 Health Research Innovation Center 2C53, Faculty of Veterinary Medicine, University of Calgary, 3330 Hospital Drive NW, Calgary, AB T2N 4N1, Canada; ana.perezcontreras@ucalgary.ca (A.P.-C.); catalina.barboza@ucalgary.ca (C.B.-S.); fathimashahnas.moham@ucalgary.ca (S.M.N.); slcheckl@ucalgary.ca (S.C.); fjvander@ucalgary.ca (F.v.d.M.); 2 Animal Health Centre, Ministry of Agriculture, Abbotsford, BC V3G 2M3, Canada; tomy.joseph@gov.bc.ca; 3 Agri Food Laboratories, Alberta Agriculture and Forestry, Edmonton, AB T6H 4P2, Canada; blking@telus.net; 4 Animal Health and Assurance, Alberta Agriculture and Forestry, Edmonton, AB T6H 4P2, Canada; madhu.ravi@gov.ab.ca (M.R.); delores.peters@gov.ab.ca (D.P.); 5 Provincial Laboratory for Public Health, Calgary, AB T2N 4W4, Canada; kevin.fonseca@albertaprecisionlabs.ca; 6 Swine and Poultry Infectious Diseases Research Center (CRIPA-Fonds de Recherche du Québec), Faculté de Médecine Vétérinaire, Université de Montréal, 3200 rue Sicotte, Saint-Hyacinthe, QC J2S 2M2, Canada; carl.a.gagnon@umontreal.ca; 7 Animal Health Laboratory, University of Guelph, Guelph, ON N1G 2W1, Canada; dojkic@uoguelph.ca

**Keywords:** infectious laryngotracheitis virus, pathogenicity, transmission, poultry, vaccine revertant infectious laryngotracheitis virus

## Abstract

Infectious laryngotracheitis (ILT) is an infectious upper respiratory tract disease that impacts the poultry industry worldwide. ILT is caused by an alphaherpesvirus commonly referred to as infectious laryngotracheitis virus (ILTV). Vaccination with live attenuated vaccines is practiced regularly for the control of ILT. However, extensive and improper use of live attenuated vaccines is related to vaccine viruses reverting to virulence. An increase in mortality and pathogenicity has been attributed to these vaccine revertant viruses. Recent studies characterized Canadian ILTV strains originating from ILT outbreaks as related to live attenuated vaccine virus revertants. However, information is scarce on the pathogenicity and transmission potential of these Canadian isolates. Hence, in this study, the pathogenicity and transmission potential of two wildtype ILTVs and a chicken embryo origin (CEO) vaccine revertant ILTV of Canadian origin were evaluated. To this end, 3-week-old specific pathogen-free chickens were experimentally infected with each of the ILTV isolates and compared to uninfected controls. Additionally, naïve chickens were exposed to the experimentally infected chickens to mimic naturally occurring infection. Pathogenicity of each of these ILTV isolates was evaluated by the severity of clinical signs, weight loss, mortality, and lesions observed at the necropsy. The transmission potential was evaluated by quantification of ILTV genome loads in oropharyngeal and cloacal swabs and tissue samples of the experimentally infected and contact-exposed chickens, as well as in the capacity to produce ILT in contact-exposed chickens. We observed that the CEO vaccine revertant ILTV isolate induced severe disease in comparison to the two wildtype ILTV isolates used in this study. According to ILTV genome load data, CEO vaccine revertant ILTV isolate was successfully transmitted to naïve contact-exposed chickens in comparison to the tested wildtype ILTV isolates. Overall, the Canadian origin CEO vaccine revertant ILTV isolate possesses higher virulence, and dissemination potential, when compared to the wildtype ILTV isolates used in this study. These findings have serious implications in ILT control in chickens.

## 1. Introduction

*Gallid herpesvirus-1* is the causative agent of the highly contagious and acute upper respiratory disease of poultry, commonly referred to as infectious laryngotracheitis (ILT) [1,2]. The ILT virus (ILTV) is an enveloped DNA virus and belongs to the family *Herpesviridae*, subfamily *Alpaherpesvirinae*, and genus *Iltovirus* [3]. Infection with this virus is mostly limited to chickens; however, occasional infection of pheasants and peafowls has been reported [4,5]. It is the cause of substantial economic losses for the poultry industry worldwide due to a decrease in body weight gain and egg production, as well as moderate to high mortality, depending on the infecting strain [1]. Clinical signs in mild presentations of the disease include watery eyes, nasal discharge, and inflammation of infraorbital sinuses and conjunctiva. In severe cases, there is depression, conjunctivitis, respiratory rales, and dyspnea with desquamation of the tracheal epithelium and expectoration of bloody mucous. There can also be presence of hemorrhagic and fibrinous exudate buildup in the tracheal lumen [5]. Epithelial cells of the tracheal mucosa and conjunctiva constitute the main site of infection. Spread of the disease may occur with secretions or droplets of infected birds having contact with the respiratory tract, oral cavity, or eyes of naïve birds [2,6]. However, it can also be transmitted through contact with vectors such as domestic and wild species of animals, insects, and fomites [7,8]. Being a herpesvirus, a characteristic feature of ILTV is the establishment of lifelong infections through latency in the infected hosts. For ILTV, latency is established in the trachea and the trigeminal ganglia [2,9,10]. Reactivation from latency to an active replication state can occur intermittently when the bird is subject to periods of stress, coinciding with events such as onset of laying, relocation, introduction of new birds to the flock, and densely populated housing environments [11].

Control of ILTV relies mainly on strict biosecurity measures and vaccination [12]. Live attenuated and recombinant vaccines are amongst the currently available and most frequently used vaccines for ILT. Attenuated vaccines originate from sequential passages of the virus on chicken embryos (CEO) or in tissue culture (TCO) [13]. On the other hand, commercially available recombinant vaccines employ turkey herpes virus (HVT) or fowl pox virus (FPV) as vectors for ILTV genes (gB, gI, and gD) [14]. Live attenuated vaccines have been broadly used over recombinant vaccines mostly due to the elicited protection, especially the CEO vaccine, which is highly effective against ILTV infections by reducing both clinical signs and viral loads in the trachea of infected birds [12]. Ease of vaccine administration to large numbers of birds is another favorable characteristic of attenuated vaccines, since they can be administered through drinking water, eye drop, or spray [15]. However, it should be highlighted that the attenuated vaccine virus will still establish latency in the vaccinated birds and can also be shed to the environment [9,11]. Most importantly, through serial passage of the vaccine virus on naïve or poorly immunized birds, the virus is likely to regain and increase virulence, prompted by recombination and mutation. This has been widely reported, especially for CEO vaccine viruses, probably related to the higher degree of attenuation of TCO vaccines making them less prone to virulence [16,17,18]. Several studies have described field-circulating ILTV strains as genetically related and, in some cases, almost indistinguishable from vaccine viruses [19,20,21,22]. Classified as vaccine revertant strains, many of these viruses lead to increased morbidity and mortality rates [16,23].

Recent studies in Canada, using partial sequences of ILTV genes (open reading frame (ORF) a and b), and using the complete ILTV genome, disclosed the dominant nature of the CEO vaccine revertant ILTV isolates [24,25]. Recombination between vaccines and wildtype viruses was also identified in these studies [25]. Studies have shown that CEO vaccine revertant ILTV strains are more pathogenic than the wildtype ILTV [26,27]. However, to date, there are no records that describe the pathogenicity and transmission potential of vaccine revertant and wildtype ILT viruses isolated in Canada. Hence, the aim of the present work was to determine the pathogenicity and transmission potential of three ILTV isolates of Canadian origin, i.e., two wildtype viruses and one CEO vaccine revertant.

## 2. Materials and Methods

### 2.1. Virus

The ILTVs used in the experimental study were isolated from backyard chicken flocks, affected with ILT and raised in province of Alberta (AB), Canada. Only samples from backyard chickens were used in the study since ILT is endemic in these flocks in Alberta and ILT outbreaks in commercial poultry operations in AB are uncommon. ILT samples (oropharyngeal swabs and trachea) positive for the ILTV genome, according to polymerase chain reaction (PCR) assays and corresponding to wildtype and CEO revertant viruses, as indicated previously [25], were obtained from the Agri Food Laboratories (Alberta Agriculture and Forestry in Edmonton, AB, Canada). The relevant history of the affected flocks has been described elsewhere [25]. In this study, we used two wildtype ILTV isolates (AB-S20 and AB-S63) and one CEO vaccine revertant ILTV isolate (AB-S45). In order to obtain adequate viral stocks to conduct experimental studies, ILTV sample AB-S63 was propagated in the chorioallantoic membrane (CAM) of 9–11 days old specific pathogen-free (SPF) chicken embryos, and isolates AB-S20 and AB-S45 were propagated in monolayers of chicken embryo liver cells (CELICs).

### 2.2. Experimental Design

Specific pathogen-free (SPF) white leghorn chickens were purchased from the Canadian Food Inspection Agency (CFIA), Ottawa, ON, Canada. The work was approved by the Health Science Animal Care Committee (HSACC) of the University of Calgary (Protocol number: AC19-0013).

At 3 weeks of age, and after 1 week of adaptation, chickens were randomly assigned to three groups (*n* = 8 per group). Chickens were infected with a dose of 10^3.5^ tissue culture infectious dose (TCID_50_) and a total volume of 200 µL per chicken, via a combination of intratracheal and intraocular routes with one of the three ILTV isolates: wildtype AB-S20, wildtype AB-S63, and CEO vaccine revertant AB-S45. Sterile phosphate-buffered saline (PBS) was administered to control chickens via intratracheal and intraocular routes in order to simulate an experimental infection. The infected chickens were kept in high containment poultry isolators (Plas Labs Inc., Lansing, MI, USA) at the Prion Virology Facility (PVF), Foothills Campus, University of Calgary. Three days post infection, three naïve contact-exposed chickens were introduced to each of the isolators and exposed for 3 days to the experimentally infected chickens. After 3 days of exposure, the contact-exposed chickens were placed in separate poultry isolators. Food and water were provided ad libitum. The control chickens were maintained at the Animal Resource Center (ARC) at Foothills Campus, University of Calgary.

Following infection, the chickens were observed for clinical manifestations. The observed clinical signs included ruffled feathers, droopy wings, conjunctivitis, depression, and respiratory signs (ranging from a mild increase in respiration to severe dyspnea). Clinical signs were assigned a score value of 1–4, depending on their severity (Table 1). Clinical signs were monitored twice a day, and three times a day on the peak of clinical sign manifestation. The experimentally infected chickens were monitored for a period of 14 days post infection (dpi), and the contact-exposed chickens were monitored for 10 days following exposure to the experimentally infected chickens. At 3, 7, 10, and 14 dpi of the experimentally infected chickens and at 3, 7, and 10 dpi of the contact-exposed chickens, cloacal and oropharyngeal swabs were taken using sterile polyester swabs in universal transport medium (Puritan, Guilford, ME, USA). After collection, swabs were stored at −80 °C until processing. At the same timepoints, bodyweights were recorded, and three flight feathers were pulled to collect the tips of the feathers in RNA Save (BI, Cromwell, CT, USA) for viral load quantification. Chickens were euthanized at 14 dpi for the experimentally infected chickens and controls and 11 dpi for the contact-exposed chickens. Postmortem examinations were done to record gross lesions. Trachea samples were collected in 10% buffered formalin for histopathological examination. In addition, oropharyngeal and cloacal swabs, as well as feather tips, were collected in RNASave for ILTV genome load quantification.

### 2.3. Techniques

#### 2.3.1. Virus Propagation on Chorioallantoic Membrane and Chicken Embryo Liver Cells (CELICs)

Virus propagation was done on a CAM as described previously [25]. For propagation of the ILTV isolates on CELICs, the livers of SPF chicken embryos on day 14 of incubation were extracted. For the preparation of CELICs, embryo livers were minced thoroughly using sterile scissors in a sterile beaker. Minced livers were rinsed three times with enough Dulbecco’s phosphate-buffered saline (DPBS) (Gibco Life Technologies, Burlington, ON, Canada) to cover the livers completely. Afterward, the remaining DPBS and minced livers were placed in a trypsinization flask (Ace Glass Inc., Vineland, NJ, USA) with trypsin/ethylenediaminetetraacetic acid (EDTA) solution at a 1:1 ratio and incubated at 37 °C on a magnetic stirrer at low speed for 15 min. Then, the trypsin/EDTA solution was filtered using a sterile cheesecloth (American Fiber & Finishing Inc., Albemarle, NC, USA) filter over a sterile beaker. Dulbecco’s modified Eagle medium (DMEM) containing 2% calf serum and 2% penicillin/streptomycin (100 U/mL penicillin and 100 µg/mL streptomycin) (Gibco Life Technologies, Burlington, ON, Canada) was then added to the filtered cell solution at a 1:1 ratio. The cell suspension was then placed into a 50 mL tube (Froggabio Inc., Toronto, ON, Canada) and centrifuged at 200× *g* for 10 min. The supernatant was discarded; the cell pellet was resuspended in growth medium after the corresponding cell count (2.1 × 10^6^) and finally seeded into T-75 tissue culture flasks (Greiner Bio One, Kremsmünster, Austria). The CELIC monolayers were inoculated with the ILTV isolates and incubated at 37 °C in 5% CO_2_ for 5 days or until the cytopathic effects were evident on 80% of the CELIC monolayer. In order to harvest the virus, the flasks were set to three freeze (−80 °C) and thaw (37 °C) cycles of 30 min each. By the end of the cycles, cells and supernatant were collected using a cell scraper (Corning, Corning, NY, USA), aliquoted in small vials, and stored at −80 °C until further use.

#### 2.3.2. Virus Titration

The CELICs were cultured in 96-well plates (U-bottom) (0.01 × 10^6^ cells per well) using DMEM containing 2% calf serum and 2% penicillin/streptomycin (Gibco Life Technologies, Burlington, ON, Canada). When the cells were 80% confluent, the propagated ILTV isolates were titrated using 10-fold serial dilution. The plates were incubated at 37 °C with 5% CO_2_. After 5 days, cytopathic effects were examined, and the titer was determined following the Reed and Muench method [28].

#### 2.3.3. DNA Purification and Quantitative Polymerase Chain Reaction (PCR) Assay

DNA purification from swabs and tissue samples (feather tips, trachea, and lungs) was carried out using the QIAmp DNeasy Kit (QIAGEN GmbH, Hilden, Mettmann, Germany) according to the manufacturer’s instructions. The purity of DNA was assessed, and quantification of DNA was done using a Nanodrop ND-1000 spectrophotometer (Thermo Scientific, Wilmington, DE, USA).

For ILTV genome quantification, a qPCR assay was carried out using a CFX96-c1000 Thermocycler (Bio-Rad laboratories, Mississauga, ON, Canada) targeting the proteinase K (PK) gene [25,29]. The total volume per reaction was 20 μL, which included genomic DNA as a template, 10 μL of SYBR Green Master Mix (Invitrogen, Burlington, ON, Canada), 0.5 μL of forward (F) and reverse (R) specific primers with a final concentration of 10 pmol/μL targeting the ILTV PK gene (F: 5’–TAC GAT GAA GCG TTC GAC TG–3’ and R: 5’–AGG CGT GAC AGT TCC AAA GT–3’), and DNAse/RNAse-free water (Thermo Scientific, Wilmington, DE, USA). Thermocycler conditions were as follows: 95 °C for 20s for initial denaturation, then 40 cycles of denaturation at 95 °C for 3 s, annealing at 60 °C for 30 s, and elongation at 95 °C for 10 s. Additionally, the β-actin genome (F: 5’–CAA CAC AGT GCT GTC TGG TGG TA–3’ and R: 5’–ATC GTA CTC CTG CTT GCT GAT CC–3’) was quantified in each of the samples to normalize the variation in template amount, using the previously mentioned PCR conditions.

#### 2.3.4. Histology

The trachea samples were submitted to the Diagnostic Services Unit (DSU) of the University of Calgary Faculty of Veterinary Medicine (UCVM) to process haematoxylin-eosin (H&E) stained sections.

### 2.4. Data and Statistical Analyses

The ILT viral load quantification was determined using standard curves of the PK gene plasmid and of β-actin, used as a housekeeping gene.

All statistical analyses in this study were carried out using GraphPad Prism 9.0.0 (GraphPad Software, San Diego, CA, USA). A two-way ANOVA and Tukey’s multiple comparison test were used to analyze bodyweight data. Survival curves were analyzed using the log rank (Mantel–Cox) test and Gehan–Breslow–Wilcoxon test. Kruskal–Wallis and Dunn’s multiple comparison tests were used for clinical score and viral genome load analyses.

## 3. Results

### 3.1. Clinical Signs

#### 3.1.1. Experimentally Infected Chickens

**Figure 1:**
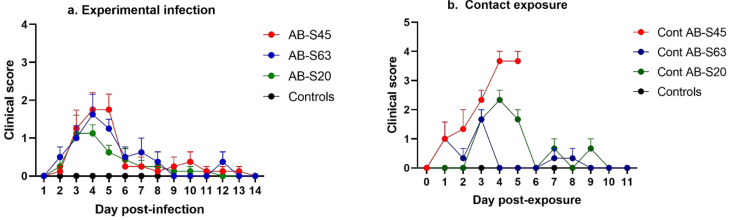
Clinical manifestations of chickens infected with two wildtype and one chicken embryo origin (CEO) vaccine revertant infectious laryngotracheitis virus (ILTV) isolates. (**a**) Clinical scores of the chickens experimentally infected with the ILTV isolates. (**b**) Clinical scores of the chickens infected via exposure to the experimentally infected chickens. Results from the contact chicken exposed to CEO revertant AB-S45 were only included until 5 dpe since the chickens were humanely euthanized at that time point in accordance with the animal use protocols. Means of the clinical scores are plotted along with bars representing standard errors. The clinical signs were scored as indicated in Section 2 and analyzed statistically using Kruskal–Wallis and Dunn’s multiple comparison tests.

The peak of clinical sign presentation was observed at 4 dpi in the three groups of infected chickens (Figure 1a), while control chickens showed no clinical signs. At 4 dpi, the differences in clinical signs between AB-S63 (*p* = 0.02), AB-S45 (*p* = 0.004), and AB-S20 (*p* = 0.04) and controls were significant. At 5 dpi, the differences in clinical signs between AB-S63 (*p* = 0.009) and AB-S45 (*p* = 0.001) and uninfected controls were significant. The clinical signs of the chickens infected with wildtype strain AB-S20 included mild but persistent inflammation of the periorbital sinuses and increased respiratory rate with occasional open-beak breathing. The chickens infected with ILTV AB-S63 developed mild inflammation of periorbital sinuses, increased respiratory rates, and difficulty breathing, manifested by open-beak respiration with characteristic extended neck posture and gasping. Clinical signs observed in some of the chickens infected with CEO revertant AB-S45 were conjunctivitis, depression, gasping, and open-beak respiration with extended neck posture. At 5 dpi, one of the chickens experimentally infected with the CEO revertant ILTV isolate surpassed the 4-point threshold established for clinical scoring system. After careful examination of the bird, it became evident that the chicken had to be humanely euthanized. The threshold was determined by the accumulation and manifestation of clinical signs considered to be incompatible with life, and used to minimize unnecessary animal suffering, adhering to the approved animal use protocol.

The histological examination of trachea originated from AB-S63, AB-S45, and AB-S20 ILTV-infected chickens at 14 dpi showed histological changes. The trachea of chickens infected with AB-S63 ILTV had epithelial metaplasia (*n* = 2) and mononuclear cell aggregates (*n* = 3). The rest of the trachea sections in AB-S63 ILTV infected chickens were normal (*n* = 3). The trachea of chickens infected with AB-S45 ILTV had mononuclear cell aggregates (*n* = 2). The rest of the trachea sections in AB-S45 ILTV infected chickens were normal (*n* = 5). The trachea of chickens infected with AB-S20 ILTV had mononuclear cell aggregates (*n* = 31). The rest of the trachea sections in AB-S20 ILTV infected chickens were normal (*n* = 7).

#### 3.1.2. Contact-Exposed Chickens

The naïve chickens exposed to the experimentally infected chickens with CEO revertant virus AB-S45 manifested severe clinical signs such as watery eyes, conjunctivitis, difficulty breathing, and constant gasping, accompanied by depression. The clinical signs peaked at 4 days post exposure (dpe) (Figure 1b). The chickens exposed to CEO vaccine revertant ILTV AB-S45 had significantly higher clinical scores compared to mock-infected controls at 3 dpe (*p* = 0.02), 4 dpe (*p* = 0.04), and 5 dpe (*p* = 0.03). The chickens exposed to CEO vaccine revertant ILTV AB-S45 had significantly higher clinical scores compared to AB-S63 at 4 dpe (*p* = 0.04) and 5 dpe (*p* = 0.03). All the chickens belonging to this group had to be humanely euthanized at 5 dpe due to a critical accumulation in their clinical scores. There were no endpoints observed for the groups of chickens infected with the ILTV wildtype strains during the course of the experiment. The naïve chickens exposed to experimentally infected chickens with wildtype viruses AB-S20 and AB-S63 displayed an increase in respiratory rates at 3, 4, and 5 dpe.

The histological examination of trachea originated from AB-S63 and AB-S45 ILTV-infected contact chickens at 11 dpi showed histological changes. The trachea of contact chickens infected with AB-S63 ILTV had debris in the lumen (*n* = 3), areas with epithelial metaplasia (*n* = 1), and areas with eroded epithelium (*n* = 3). The trachea of contact chickens infected with AB-S45 ILTV had debris in the lumen (*n* = 3), areas with epithelial metaplasia (*n* = 2), and areas with eroded epithelium (*n* = 1). The trachea of contact chickens infected with AB-S20 ILTV were normal (*n* = 3).

### 3.2. Bodyweights

#### 3.2.1. Experimentally Infected Chickens

**Figure 2:**
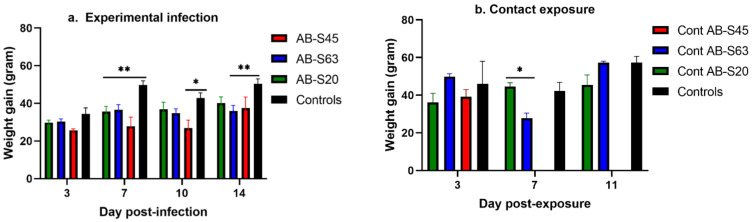
Weight gains of chickens infected with two wildtype and one CEO vaccine revertant ILTV isolates. The infected and control chickens were weighed on days 0, 3, 7, 10, or 11, and 14 following ILTV infection, and weight gains were calculated. Means of the weight gains are plotted along with bars representing standard errors. Two-way ANOVA and Tukey’s multiple comparison tests were used to identify significance. (**a**) Average weight gains of experimentally infected chickens and mock-infected controls. (**b**) Weight gains of chickens infected via contact exposure and unexposed controls. Weight gains from the contact chicken exposed to CEO revertant AB-S45 were not included after 3 dpe due to humane euthanasia of the chickens at 5 dpe, in accordance with the animal use protocols. * = *p* < 0.05, ** = *p* < 0.001.

Overall, bodyweight gains in the chickens experimentally infected with the two wildtype ILTVs were similar and remained constant throughout the entire experiment (Figure 2a). At 7 dpi, significant differences were found in weight gains between uninfected controls and chickens infected with wildtype AB-S63 (*p* = 0.009), CEO vaccine revertant AB-S45 (*p* = 0.009), and wildtype AB-S20 *(p* = 0.0058). At 10 dpi, the AB-S45 ILTV-infected group had significantly lower weight gains compared to the mock-infected controls (*p* = 0.03). At 14 dpi, the AB-S63 ILTV-infected group had significantly lower weight gains compared to the mock-infected controls (*p* = 0.009).

#### 3.2.2. Contact-Exposed Chickens

At 3 dpe, the chickens exposed to experimentally infected chickens with vaccine revertant ILTV AB-S45 and wildtype ILTVs AB-S63 and AB-S20 had similar weight gains to the controls (*p* > 0.05) (Figure 2b). It is worth mentioning that, by 5 dpe, when the chickens exposed to AB-S45 ILTV reached the humane endpoints, two of the exposed chickens had lost on average 12% of their initial bodyweight, and the third chicken had gained little to no weight. At 7 dpe, the differences in weight gains between AB-S20- and AB-S63-exposed groups were significant (*p* = 0.03).

### 3.3. Survival Rate

#### 3.3.1. Experimentally Infected Chickens

**Figure 3:**
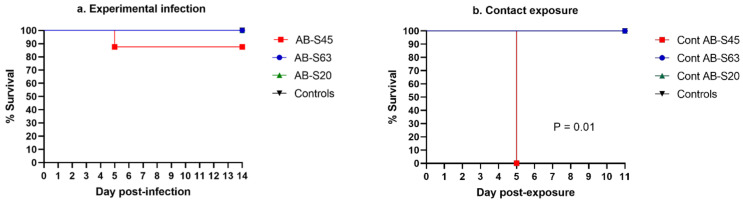
Survival percentage of chickens infected with two wildtype and one CEO vaccine revertant ILTV isolates. (**a**) Percentage survival of experimentally infected chickens. (**b**) Percentage survival of chickens infected via contact exposure. Following ILTV infection, the chickens were scored for development of clinical signs in order to determine the humane endpoints. The chickens that reached the endpoints were euthanized, and the percentage of surviving chickens was recorded. The survival rates of the groups were compared to identify significant differences using the log rank (Mantel–Cox) test and Gehan–Breslow–Wilcoxon test.

The survival rate of experimentally infected chickens and their uninfected controls is illustrated in Figure 3a. Only one chicken reached a humane endpoint, at 5 dpi in the group of chickens infected with vaccine revertant ILTV AB-S45.

#### 3.3.2. Contact-Exposed Chickens

The survival rate of chickens exposed to experimentally infected chickens and their unexposed controls is illustrated in Figure 3b. The chickens exposed to vaccine revertant ILTV AB-S45 had a survival rate of 0% by 5 dpe. The chickens in the other groups did not reach the endpoints before the established end of the experiment at 11 dpe. The survival rate between vaccine revertant ILTV AB-S45 and the other groups was significantly different (*p* = 0.01).

### 3.4. Viral Genome Loads

#### 3.4.1. Oropharyngeal Swabs

##### Experimentally Infected Chickens

**Figure 4:**
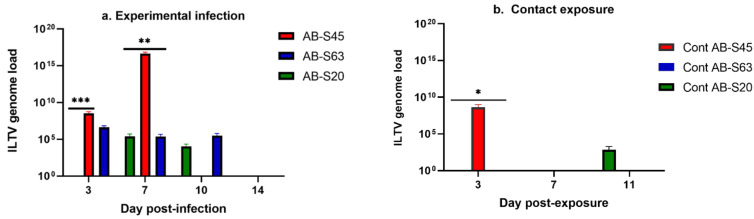
The viral genome loads quantified in oropharyngeal swabs of chickens infected with two wildtype and one CEO vaccine revertant ILTV isolates. Means of the viral genome loads in log_10_ scale are plotted with bars representing standard errors. ILTV genome loads were quantified by targeting the proteinase K (PK) gene using the SYBR Green method. Kruskal–Wallis and Dunn’s multiple comparison tests were used for group comparisons. (**a**) ILTV genome loads of the experimentally infected chickens. (**b**) ILTV genome loads of chickens infected via contact exposure. Viral genome loads from the contact chicken exposed to CEO revertant AB-S45 were not included after 3 dpe due to humane euthanasia of the chickens at 5 dpe, in accordance with the animal use protocols. * = *p* < 0.05, ** = *p* < 0.001, *** = *p* < 0.0001.

The ILTV genome loads in oropharyngeal swabs of experimentally infected chickens are illustrated in Figure 4a. At 3 dpi, the genome load in the ILTV AB-S45-infected group was significantly higher than that in the ILTV AB-S20-infected group (*p* = 0.0001). At 7 dpi, the genome load in the ILTV AB-S45-infected group was significantly higher than that in the groups experimentally infected with ILTV AB-S20 (*p* = 0.009) and ILTV AB-S63 (*p* = 0.007). However, no viral load was detected in oropharyngeal swabs of the ILTV AB-S45-infected groups at 10 and 14 dpi.

##### Contact-Exposed Chickens

The ILTV genome loads in oropharyngeal swabs of chickens exposed to experimentally infected chickens are illustrated in Figure 4b. No ILTV genome in oropharyngeal swabs was quantified in chickens exposed to chickens experimentally infected with wildtype ILTV AB-S63. In the group of chickens exposed to the chickens experimentally infected with wildtype ILTV AB-S20, the virus was recovered only from the oropharyngeal swabs of one of the chickens at 10 dpe. All the chickens exposed to the chickens experimentally infected with CEO vaccine revertant AB-S45 had detectable viral loads at 3 dpe in oropharyngeal swabs, which were significantly higher compared to the ILTV genome loads quantified in groups exposed to AB-S63 (*p* = 0.03) and AB-S-20 (*p* = 0.03).

#### 3.4.2. Cloacal Swabs

##### Experimentally Infected Chickens

**Figure 5:**
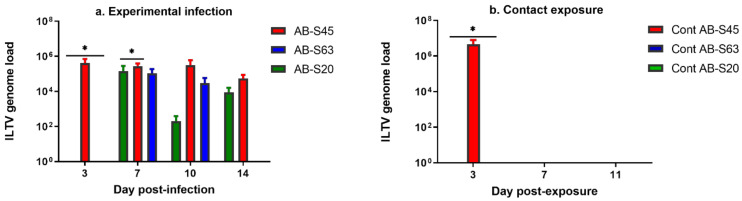
The viral genome loads quantified in cloacal swabs of chickens infected with two wildtype and one CEO vaccine revertant ILTV isolates. Means of the viral genome loads in log_10_ scale are plotted with bars representing standard errors. ILTV genome loads were quantified by targeting the proteinase K (PK) gene using the SYBR Green method. Kruskal–Wallis and Dunn’s multiple comparison tests were used for group comparisons. (**a**) ILTV genome loads of the experimentally infected chickens. (**b**) ILTV genome loads of chickens infected via contact exposure. Genome loads belonging to the contact chicken exposed to CEO revertant AB-S45 were not included after 3 dpe due to humane euthanasia of the chickens at 5 dpe, in accordance with the animal use protocols. * = *p* < 0.05.

The ILTV genome loads in cloacal swabs of experimentally infected chickens are illustrated in Figure 5a. At 3 dpi, the genome load in cloacal swabs of chickens experimentally infected with ILTV AB-S45 was significantly higher compared to that in chickens experimentally infected with ILTV AB-S20 (*p* = 0.020) and AB-S63 *(p* = 0.020). At 7 dpi, the genome load in cloacal swabs of chickens experimentally infected with ILTV AB-S45 was significantly higher compared to that in chickens experimentally infected with ILTV AB-S20 (*p* = 0.020).

##### Contact-Exposed Chickens

The ILTV genome loads in cloacal swabs of chickens exposed to experimentally infected chickens are illustrated in Figure 5b. All chickens exposed to the chickens experimentally infected with CEO vaccine revertant AB-S45 had quantifiable genome loads in cloacal swabs at 3 and 7 dpe, and they were significantly higher compared to those in groups infected with ILTV AB-S20 (*p* = 0.050) and AB-S63 *(p =* 0.050). In fact, no virus was recovered from the cloacal swabs of the chickens exposed to chickens experimentally infected with wildtype ILTV AB-S20 and AB-S63.

#### 3.4.3. Feathers

##### Experimentally Infected Chickens

**Figure 6:**
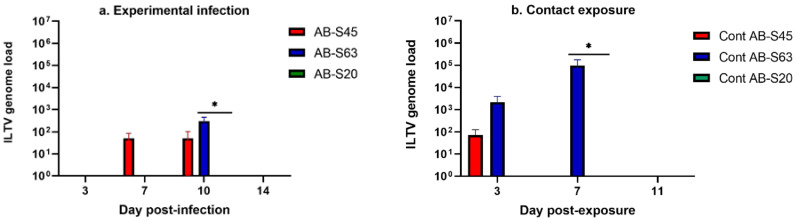
The viral genome loads quantified in feathers of chickens infected with two wildtype and one CEO vaccine revertant ILTV isolates. Means of the viral genome loads in log_10_ scale are plotted with bars representing standard errors. ILTV genome loads were quantified by targeting the proteinase K (PK) gene using the SYBR Green method. Kruskal–Wallis and Dunn’s multiple comparison tests were used for group comparisons. (**a**) ILTV genome loads of the experimentally infected chickens. (**b**) ILTV genome loads of chickens infected via contact exposure. Viral genome loads from the contact chicken exposed to CEO revertant AB-S45 were not included after 3 dpe due to humane euthanasia of the chickens at 5 dpe, in accordance with the animal use protocols. * = *p* < 0.05.

The ILTV genome loads in feathers of experimentally infected chickens are illustrated in Figure 6a. We could not recover the ILTV genome from the feathers of chickens experimentally infected with wildtype ILTV AB-S20. At 10 dpi, the genome load in feather tips of chickens experimentally infected with ILTV AB-S63 was significantly higher compared to that in chickens experimentally infected with ILTV AB-S20 (*p* = 0.01).

##### Contact-Exposed Chickens

The ILTV genome loads in feathers of contact exposed chickens are illustrated in Figure 6b. We could not recover the ILTV genome from the feathers of chickens exposed to chickens experimentally infected wildtype ILTV AB-S20. At 7 dpe, the genome load in feather tips of chickens exposed to chickens experimentally infected with ILTV AB-S63 was significantly higher compared to that in chickens exposed to chickens experimentally infected with ILTV AB-S20 (*p* = 0.01).

#### 3.4.4. Trachea and Lungs

##### Experimentally Infected Chickens

**Figure 7:**
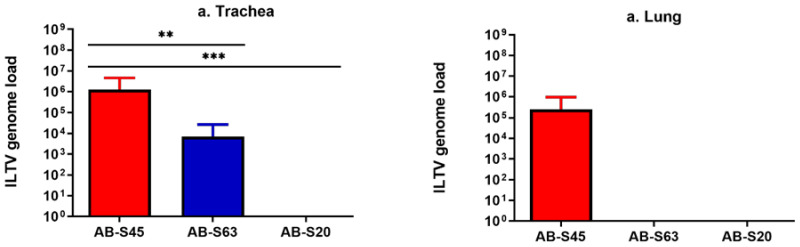
The viral genome loads quantified in the trachea and lungs of chickens experimentally infected with two wildtype and one CEO vaccine revertant ILTV isolates, sampled at 14 days post infection. Means of the viral genome loads in log_10_ scale are plotted with bars representing standard errors. ILTV genome loads were quantified by targeting the proteinase K (PK) gene using the SYBR Green method. Kruskal–Wallis and Dunn’s multiple comparison tests were used for group comparisons. (**a**) ILTV genome loads in the trachea. (**b**) ILTV genome loads in the lungs. ** = *p* < 0.001, *** = *p* < 0.0001.

The ILTV genome loads in the trachea and lungs of experimentally infected chickens are illustrated in Figure 7a,b. At the end of the experiment (14 dpi), no ILTV genome was quantifiable from the trachea and lungs originating from chickens experimentally infected with wildtype ILTV AB-S20. At the same timepoint, the genome load in the trachea of chickens experimentally infected with ILTV AB-S45 was significantly higher compared to that in chickens experimentally infected with ILTV AB-S20 (*p* = 0.0002) and AB-S63 (*p* = 0.001). In lungs, the ILTV genome was quantifiable only from the chickens experimentally infected with ILTV AB-S45, and the group differences were not different significantly (*p* > 0.05).

##### Contact-Exposed Chickens

At the end of the experiment (11 dpi for contact-exposed chickens), no ILTV genome was quantifiable from the trachea or lungs originating from chickens exposed to the experimentally infected chickens with wildtype ILTV AB-S20 and AB-S63. Since all the chickens exposed to chickens experimentally infected with CEO vaccine revertant ILTV AB-S45 reached the endpoints at 5 dpe, we quantified the ILTV genome loads at this point in the trachea and lungs and found that the ILTV genome was quantifiable from the trachea (median, 2 × 10^5^) and lungs (median, 1.4 × 10^4^) of all three contact-exposed chickens.

## 4. Discussion

Although ILT control relies heavily on vaccination using live attenuated vaccines, vaccine revertant ILTV strains are increasingly gaining attention due to their involvement in ILT outbreaks. The previous work conducted in Canada by us and others, provided evidence of circulating ILTVs genetically related to live attenuated vaccine strains [24,25,30]. In fact, the proportion of CEO vaccine revertant ILTV isolates was higher (71%) than wildtype ILTV isolates (21%) in the aforementioned studies. This observation is intriguing, and we hypothesized that CEO vaccine revertant ILTV isolates possess higher pathogenicity and transmission potential, leading to their dominance in poultry flocks. Therefore, the aim of this study was to compare the pathogenicity and transmission potential of three ILTV isolates originating in Alberta, Canada [24]. Two of them were characterized as wildtype, and one of them as a CEO vaccine revertant ILTV isolate.

Compared to the wildtype ILTV, the CEO vaccine revertant ILTV had a higher pathogenicity potential, as shown by the higher clinical scores, tracheal pathology, significantly lower weight gains, and lower survival rate. Our observation in this regard agrees with previous studies [16,23,27], where the severity of clinical manifestations and an increase in mortality rates were constant characteristics observed in chickens infected with CEO revertant ILTV isolates.

We evaluated the transmission potential of ILTV using contact exposure of naïve chickens to experimentally infected chickens, as has been done previously [17,31]. The results of this work reveal a greater transmission potential of the CEO vaccine revertant ILTV isolate (AB-S45) when compared to the wildtype ILTV. While the wildtype ILTV was cleared from the respiratory tract by the end of the study (14 dpi), the CEO vaccine revertant ILTV isolate AB-S45 persisted in the trachea and lungs, as evidenced by ILTV genome loads in swabs and tissues, and histological changes in trachea [31]. In this study, we did not investigate why the CEO vaccine revertant had higher transmission potential, but we believe that it may be due to the shedding of a higher ILTV amount and/or a higher number of routes, as shown by the results of this study. These results could account for the dominance of the CEO vaccine revertant ILTV in the field. Nevertheless, we cannot generalize these conclusions to all ILTV vaccine revertant strains in Canada on the basis of the present findings, which are variable. Future studies evaluating the pathogenicity and transmission potential of other ILTV vaccine revertant strains from AB and other provinces of Canada are required to confirm the present observation. Another interesting finding that may be relevant to the transmission potential is that only the CEO vaccine revertant ILTV genome persisted in the respiratory tract until 14 dpi, unlike the wildtype ILTV. Whether this observation has any implication in transmission potential requires further investigations.

An additional and important finding of our study is that two out of the three tested ILTV isolates targeted the feather follicles, providing evidence that some ILTV strains could be shed to the environment via feather dander, in addition to oropharyngeal and cloacal routes.

Historically, ILTV is known to confine its replication to the respiratory tract tissues; however, it has now been shown that certain strains of ILTV can replicate in multiple body organ systems such as the respiratory tract, gastrointestinal tract, skin, and immune system organs, excreting the virus into the environment via feces and feather dander, in addition to respiratory and oropharyngeal secretions [26,32,33]. In agreement with the observation of Davidson and colleagues [32], we also found that some wildtype and CEO vaccine revertant ILTVs could replicate in feather tips, but at a lower rate. This observation implies that viral replication in feather tips may add another route of shedding of ILTV to the environment. Although we did not observe the presence of ILTV in feather dander, the possibility of the presence of the ILTV genome in feather dust was shown [34].

In conclusion, ILTV isolate AB-S45, a CEO vaccine revertant, originating from backyard chickens in Alberta, Canada, has a higher pathogenicity and transmission potential when compared to wildtype ILTV isolates AB-S63 and AB-S20. However, further studies are required to confirm our observations and to see if the CEO vaccine revertant is controllable using currently available ILT vaccines.

## Figures and Tables

**Table 1:** Clinical signs and assigned score. Chickens with an accumulative score of 4 were eligible for humane euthanasia according to the established animal use protocol.

Clinical Manifestation	Score (1–4)
Ruffled feathers	1
Droopy wings	1
Depression	1
Bodyweight loss (5%)	1
Increased respiratory rate	1
Increased respiratory rate with open beak	2
Conjunctivitis	2
Severe increase in respiratory rate marked by gasping	3
Bloody mucus expectoration	4

## Data Availability

The data presented in this study are available on request from the corresponding author.
